# The efficacy of hypothermia combined with thrombolysis or mechanical thrombectomy on acute ischemic stroke: a systematic review and meta-analysis

**DOI:** 10.3389/fneur.2024.1481115

**Published:** 2025-01-07

**Authors:** Dan Wang, Dan Yan, Mingmin Yan, Hao Tian, Haiwei Jiang, Bifeng Zhu, Yu Chen, Tao Peng, Yue Wan

**Affiliations:** Department of Neurology, Hubei No. 3 People’s Hospital of Jianghan University, Wuhan, China

**Keywords:** therapeutic hypothermia, selective cerebral hypothermia, acute ischemic stroke, thrombolysis, mechanical thrombectomy, mRS

## Abstract

**Background:**

Therapeutic hypothermia improves outcomes in experimental stroke models, especially after ischemia-reperfusion injury. In recent years, the safety and efficacy of hypothermia combining thrombolysis or mechanical thrombectomy have attracted widespread attention. The primary objective of the study was to evaluate the effectiveness and safety of hypothermia by combining reperfusion therapy in acute ischemic stroke patients.

**Methods:**

A systematic search was performed in PubMed, EMBASE, Cochrane Library, and the Clinical Trial Registries on articles published until May 2024. The full-text articles were thoroughly reviewed, and relevant information regarding study characteristics and outcomes was extracted. Mantel–Haenszel (M–H) random-effects model was used to calculate pooled risk ratios (RR) with 95% confidence intervals (CI). In addition, subgroup analyses were performed focusing on the different hypothermia modalities and duration.

**Results:**

After screening 2,265 articles, 10 studies were included in the present analysis with a total sample size of 785. Forest plots of clinical outcomes were as follows: modified Rankin Scale (mRS) ≤2 at 3 months (RR = 1.28, 95% CI 1.01–1.61, *p* = 0.04), mortality within 3 months (RR = 0.95, 95% CI 0.69–1.29, *p* = 0.73), total complications (RR = 1.02, 95% CI 0.89–1.16, *p* = 0.77) and pneumonia (RR = 1.35, 95% CI 0.76–2.40, *p* = 0.31). Subgroup analyses indicated a mild protective effect of selective cerebral hypothermia; however, the difference in mortality between the hypothermia and control groups was not statistically significant (RR = 0.88, 95% CI 0.57–1.35, *p* = 0.55). Patients undergoing hypothermia for 24–48 h experienced a higher rate of overall complications (RR = 1.37, 95% CI 1.01–1.86, *p* = 0.04) and pneumonia (RR = 2.84, 95% CI 1.05–7.66, *p* = 0.04).

**Conclusion:**

The preliminary evidence supports the safety and feasibility of hypothermia combined with reperfusion therapy, which should be further investigated in randomized controlled studies.

**Systematic review registration:**

https://www.crd.york.ac.uk/prospero/, identifier CRD42024556625.

## 1 Introduction

Acute ischemic stroke (AIS) remains one of the most significant challenges in global health, standing as a leading cause of mortality and long-term disability worldwide (1). Following the initial trauma, individuals suffering from AIS may be subjected to secondary injury and infarct growth as a result of detrimental processes such as excitotoxicity, blood-brain barrier disruption, and peri-infarct depolarization (2). Despite prompt reperfusion therapy, these factors may still contribute to cerebral edema and exacerbate outcomes. Therapeutic hypothermia (TH) is effective and safe in patients with global cerebral ischemia induced by cardiac arrest or neonatal hypoxic-ischemic encephalopathy (3, 4). Additionally, recent research (5) indicates hypothermia may offer neuroprotection by inhibiting the above-mentioned harmful processes. TH can be induced via two primary methods: whole-body cooling or targeted reduction of brain temperature while maintaining normal body temperature (6). The more prevalent approach, systemic hypothermia (systTH), can be induced through a variety of techniques such as external surface cooling, intravenous infusions of cooled saline, or the placement of specialized cooling devices in the inferior vena cava (7). Selective cerebral hypothermia (selTH) can be achieved through external surface cooling of the head or neck, the introduction of cooled saline into the nasopharynx via a specialized balloon, or the insertion of a catheter into the carotid artery or intracranial cavity (8, 9). Recently, the efficacy and safety of combined TH and thrombolysis or mechanical thrombectomy in AIS patients have attracted attention. Some studies (6, 10) have found a higher rate of side effects in patients with AIS after hypothermia, while others (11, 12) have shown no difference in adverse effects between patients treated with hypothermia and conventional treatment. Furthermore, an increasing number of studies (5, 11) suggested that TH enhanced good neurological outcomes in ischemic stroke patients. The efficacy and safety of combining hypothermia with reperfusion therapy remained inadequately substantiated. Consequently, we performed this meta-analysis to assess the clinical efficacy, safety, and potential clinical application of TH.

## 2 Methods

The protocol of this study is available in PROSPERO (International Prospective Register of Systematic Reviews, registration code: CRD42024556625) and follows the Preferred Reporting Items for Systematic Reviews and Meta-analyses for Protocols Statement (PRISMA-P) (13).

### 2.1 Search strategy

Two authors (DW and DY) independently included all the relevant studies by searching PubMed, EMBASE, Cochrane Central Register of Controlled Trials, and ClinicalTrials.gov from the inception of these sources until May 2024. The search employed keywords such as “hypothermia” and “stroke,” along with Medical Subject Heading (MeSH) terms, synonyms, and Boolean operators. Additionally, we searched the reference lists of the included articles for potential relevant literature. The detailed search strategy is shown in Supplementary material S1.

### 2.2 Inclusion criteria

(1) Studies published before May 2024. (2) Patients in the studies must have received recanalization therapies such as thrombolysis or mechanical thrombectomy, by the latest guidelines for the diagnosis and treatment of AIS (14). (3) Research must investigate the curative effect and prognosis of therapeutic hypothermia in AIS patients. (4) Full text must be accessible.

### 2.3 Exclusion criteria

(1) Animal experiments, cellular studies, and other laboratory research. (2) Comments, letters, reviews, and conference abstracts. (3) Studies that lack sufficient information to extract risk ratios (RR) or hazard ratios (HR). (4) Studies that utilize duplicated data. (5) Studies published in languages other than English.

### 2.4 Study selection

Two authors (DW and DY) independently conducted the screening of the research literature; in the case of a disagreement, this was discussed with a third author (HT) and resolved. Titles and abstracts of all articles were initially screened, and then full texts were carefully assessed according to the inclusion and exclusion criteria. Eligible studies met the following PICOS criteria: (1) Population: AIS patients who received thrombolysis or mechanical thrombectomy. (2) Intervention: utilization of hypothermia treatment. (3) Comparison intervention: normothermia. (4) Outcome: neurological outcome and survival. (5) Study design: randomized controlled trials (RCTs) or observational cohort trials (OCTs).

### 2.5 Information extraction and study endpoints

Two authors (DW and MY) extracted the data from the included trials independently on patient characteristics, details of hypothermia such as type and duration, and clinical outcomes.

In cases of missing or incomplete data, we will attempt to contact the corresponding authors to obtain the relevant information. The primary outcome was a good functional outcome at 3 months poststroke, defined as a modified Rankin Scale (mRS) Score of 0–2. Secondary outcomes included total complications, including intracerebral hemorrhage (symptomatic, asymptomatic, hemorrhagic transformation), urinary tract infection, abnormal blood coagulation, vasospasm, deep vein thrombosis, cardiac complications (hypotension/ hypertension, bradycardia/tachycardia, myocardial infarction, congestive heart failure, and arrhythmia), pneumonia, and mortality within 3 months.

### 2.6 Risk of bias assessment

The risk of bias evaluation was performed according to the Cochrane Handbook for Systematic Reviews of Interventions. We assessed the seven items separately (Supplementary material S2). However, due to the nature of the intervention, blinding of participants and personnel tended to have a high risk of bias. Under each domain, studies were classified as low, high, or unclear risk of bias. In addition, a funnel plot was constructed and Egger’s test and trim-and-fill analysis was conducted to detect the presence of potential publication bias in this random-effects meta-analysis model using statistical software Stata /MP version 18.0 (Stata Corp LLC, College Station, United States).

### 2.7 Statistical analysis

Mantel–Haenszel random-effects model was used to calculate pooled risk ratios (RR) with 95% confidence intervals (CI). The heterogeneity was evaluated by Cochran’s *Q*-statistic test and *I*-squared (*I*^2^). An *I*^2^-value of greater than 50% was considered to indicate substantial heterogeneity. Statistical significance was set at the two-tailed level of 0.05 for hypothesis testing of effect and 0.10 for hypothesis testing of heterogeneity. Statistical analyses were performed using RevMan 5.4.1 and Stata 18.0 software. Analyses followed an intention-to-treat principle using all available data.

## 3 Results

### 3.1 Literature search

**Figure 1:**
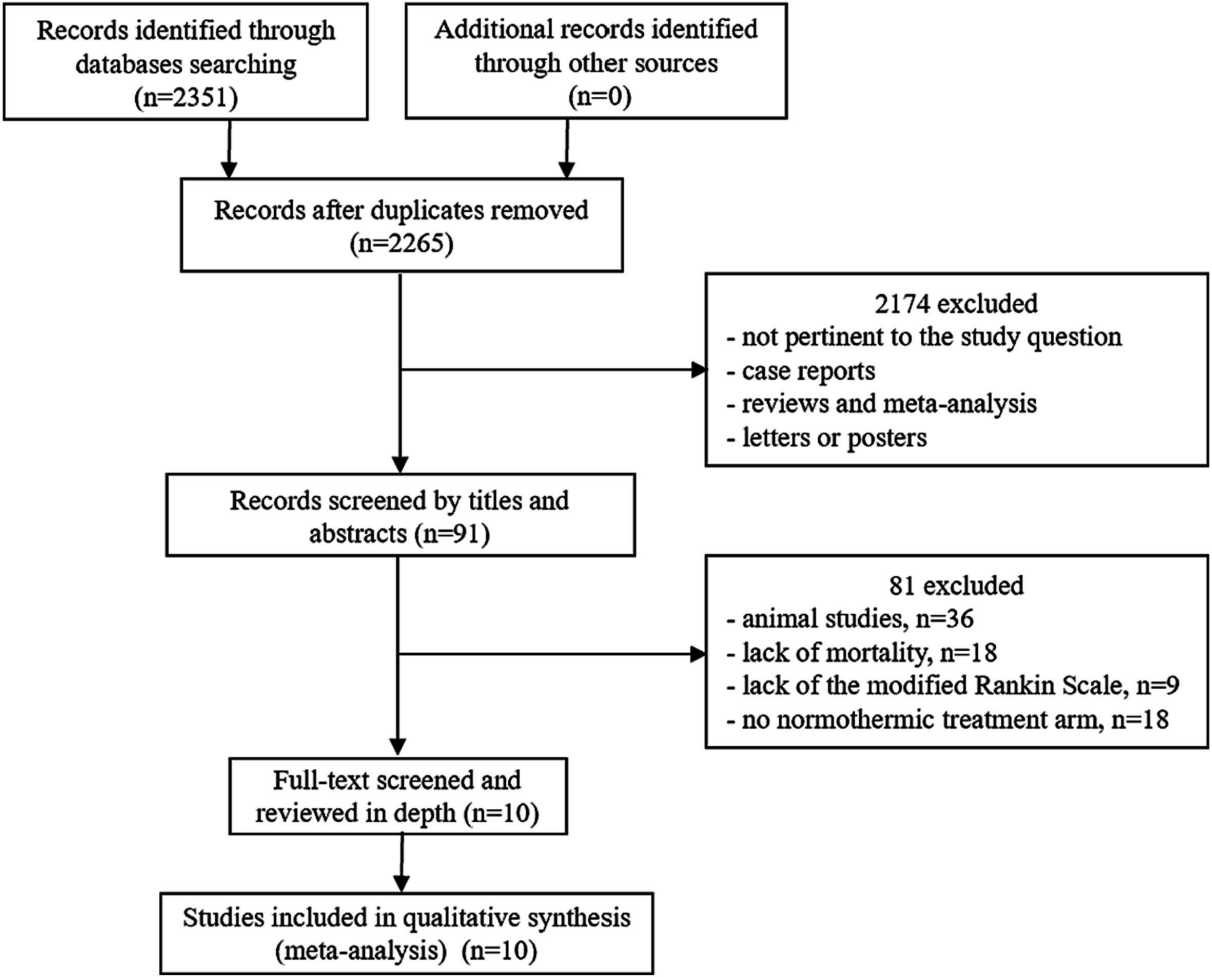
Study flow chart. After excluding 86 repetitive literature works, 2,265 studies were preliminarily screened. Based on titles and abstracts, 2,174 citations were excluded. In the remaining 91 articles, 81 articles with animal studies or incomplete data were further excluded after evaluation of the full text. Finally, a total of 10 citations were included in this meta-analysis.

Database searches and reference lists yielded a total of 2,351 articles. After deleting 86 duplicate articles, we screened the remaining 2,265 records according to the title and abstract, and 2,174 additional articles were excluded. Of the remaining 91 articles, 81 articles were further excluded after full-text evaluation. Overall, 10 articles with 785 patients were selected for inclusion and final data extraction (5, 7, 9, 11, 12, 15–19). The flowchart of the literature search flow is presented in Figure 1.

### 3.2 Study characteristics

Six RCTs and four OCTs were identified in the present analysis. Six studies involved multiple centers, and a total of 785 patients with acute ischemic stroke (*n* = 364 TH, median age: 68 years; *n* = 421 controls, median: 69 years) were included. The baseline NIHSS scores were not significantly different between patients in the control group and those with TH [median (IQR); 15 (14–16) versus 17 (13–17), *p* = 0.423]. All included studies reported the number of patients with good neurological outcomes (mRS ≤2 at 3 months), as well as the number of deaths within the same timeframe. Nine studies documented post-treatment complications, while eight studies provided specific counts of pneumonia cases. The characteristics of TH groups and control groups are summarized in Table 1.

**Table 1:** Characteristics of included studies.

References	Publish year	Country	Total complications (yes/no)	Pneumonia (yes/no)	Outcome (survival/died)	mRS ≤2 (yes/no)	Baseline NIHSS	Study method	Case (*n*)
Bi et al. (7)	2011	China	H 7/24	NA	H 27/4	H 13/18	H 11	RCT	62
			C 7/24	NA	C 28/3	C 11/20	C 11		
Choi et al. (15)	2021	Korea	H 16/12	NA	H 22/6	H 9/19	H 17	RCT	80
			C 27/25	NA	C 37/15	C 3/49	C 16		
Hemmen et al. (12)	2010	USA	H 21/7	H 14/14	H 22/6	H 5/23	H 14	RCT	58
			C 13/17	C 3/27	C 25/5	C 7/23	C 14		
Hong et al. (16)	2014	Korea	H 11/28	H 2/37	H 33/6	H 19/20	H 17	OCT	75
			C 17/19	C 11/25	C 31/5	C 8/28	C 16		
Krieger et al. (11)	2001	USA	H 9/1	H 3/7	H 7/3	H 5/5	H 20	OCT	19
			C 7/2	C 1/8	C 7/2	C 1/8	C NA		
Li et al. (17)	2022	China	H 8/32	H 4/36	H 38/2	H 26/14	NA	RCT	80
			C 14/26	C 4/36	C 36/4	C 19/21	NA		
Lyden et al. (18)	2016	USA	H 26/37	H 12/51	H 53/10	H 21/42	H 14	RCT	120
			C 20/37	C 6/51	C 52/5	C 22/35	C 15		
Piironen et al. (19)	2014	Australia	NA	H 7/11	H 18/0	H 7/11	H 11	RCT	36
			NA	C 2/16	C 16/2	C 7/11	C 14		
Tian et al. (5)	2023	China	H 45/17	H 15/47	H 48/14	H 34/28	H 17	OCT	142
			C 62/18	C 17/63	C 60/20	C 38/42	C 15		
Wu et al. (9)	2018	China	H 44/1	H 14/31	H 36/9	H 23/22	H 17	OCT	113
			C 66/2	C 23/45	C 50/18	C 28/40	C 16		

mRS, modified Rankin Scale; H, hypothermia group; C, control group; OCT, observational cohort trial; RCT, randomized controlled trial; NA, not available.

Table 2 provides an overview of the hypothermia treatment characteristics. The median time from the appearance of symptoms to TH initiation was 6 h. The mode of inducing hypothermia was highly variable, including one systemic endovascular, two systemic surfaces, three selective, and four systemic combined. Seven studies utilizing systTH reported the median target temperature for hypothermia treatment was 34.5°C, ranging from 32 to 35°C. The goal temperature was achieved within a median of 3.5 h (range 0.28–6.3 h) after the initiation of TH, and the average duration of hypothermia treatment was 46 h, ranging from 24 to 144 h.

**Table 2:** Hypothermia characteristics of included studies.

References	TH onset from stroke symptoms (h)	Type of TH	Target temperature (°C)	Duration of TH (h)	Time to reach the target (h)	Rewarming rate (°C/h)	Duration of dewarming (h)
Bi et al. (7)	6.0	Selective	NA	24	0.28	NA	NA
Choi et al. (15)	NA	Systemic combined	34.5	48	NA	0.50/12	NA
Hemmen et al. (12)	6.0	Systemic endovascular	33	24	2.3	NA	NA
Hong et al. (16)	3.0	Systemic combined	34.5	48	6.3	0.50/12	48
Krieger et al. (11)	6.2	Systemic surface	32	48	3.5	0.21/1	23
Li et al. (17)	NA	Systemic surface	33–35	144	NA	0.20/1	20
Lyden et al. (18)	3.0	Systemic combined	33	24	NA	NA	12
Piironen et al. (19)	6.0	Systemic combined	35	12	4.5	0.20/1	7
Tian et al. (5)	NA	Selective	NA	0.15	NA	NA	NA
Wu et al. (9)	5.7	Selective	NA	0.17	NA	NA	NA

TH, therapeutic hypothermia; NA, not available. TH onset from stroke symptoms, time to reach the target temperature and duration of rewarming were shown as median values.

### 3.3 Quantitative data synthesis

**Figure 2:**
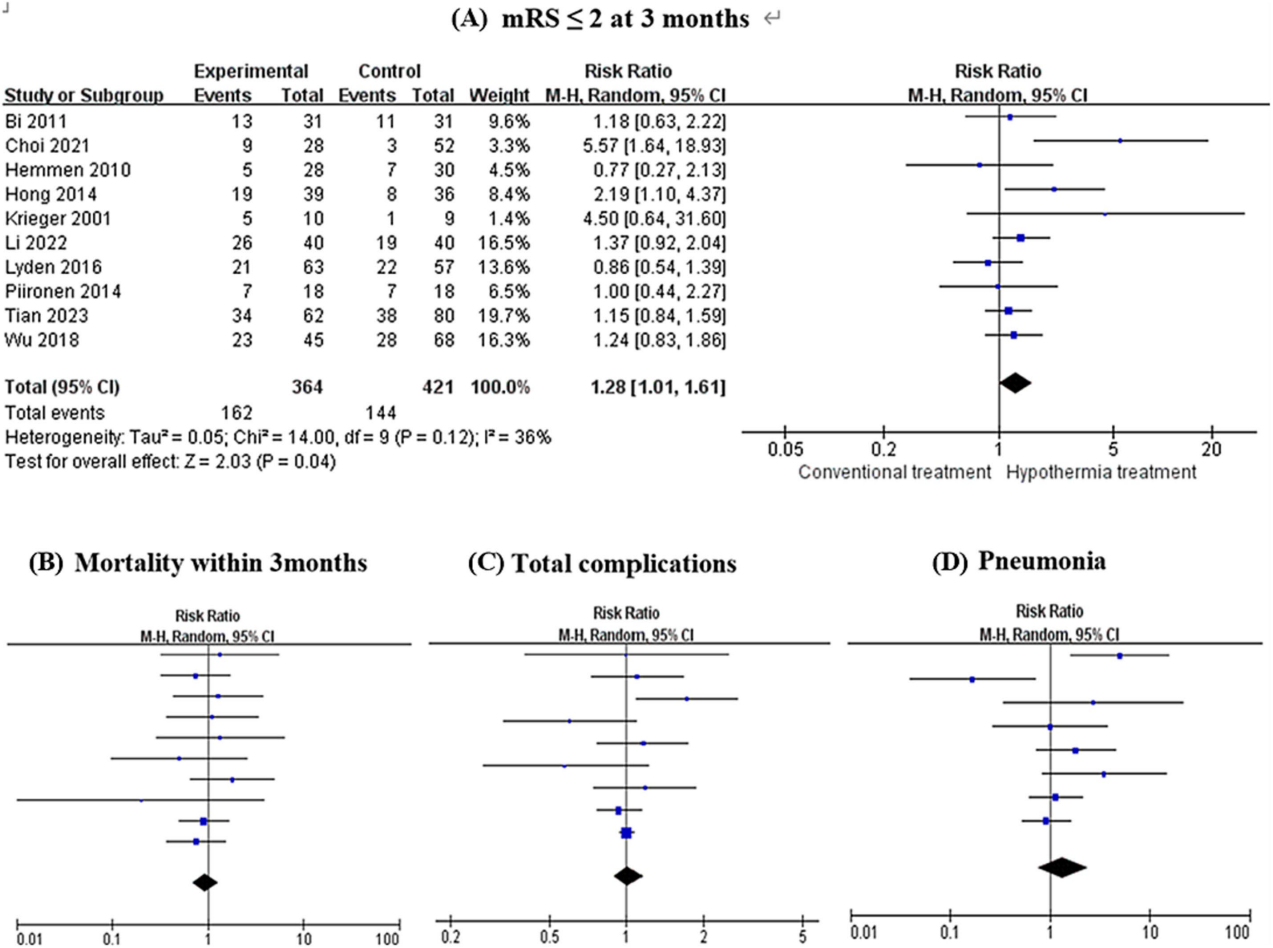
Forest plots evaluating the association of therapeutic hypothermia and clinical outcomes. There was a statistically significant difference in the primary outcome between the hypothermia treatment groups and the control groups (RR = 1.28, 95% CI 1.01–1.61, *I*^2^ = 36%, *p* = 0.04) **(A)**. Additionally, hypothermia treatment was not associated with an increased risk of pneumonia (RR = 1.35, 95% CI 0.76–2.40, *I*^2^ = 61%, *p* = 0.31) **(D)**, total complications (RR = 1.02, 95% CI 0.89–1.16, *I*^2^ = 33%, *p* = 0.77) **(C)**, and mortality within 3 months (RR = 0.95, 95% CI 0.69–1.29, *I*^2^ = 0.00%, *p* = 0.73) **(B)**.

**Figure 3:**
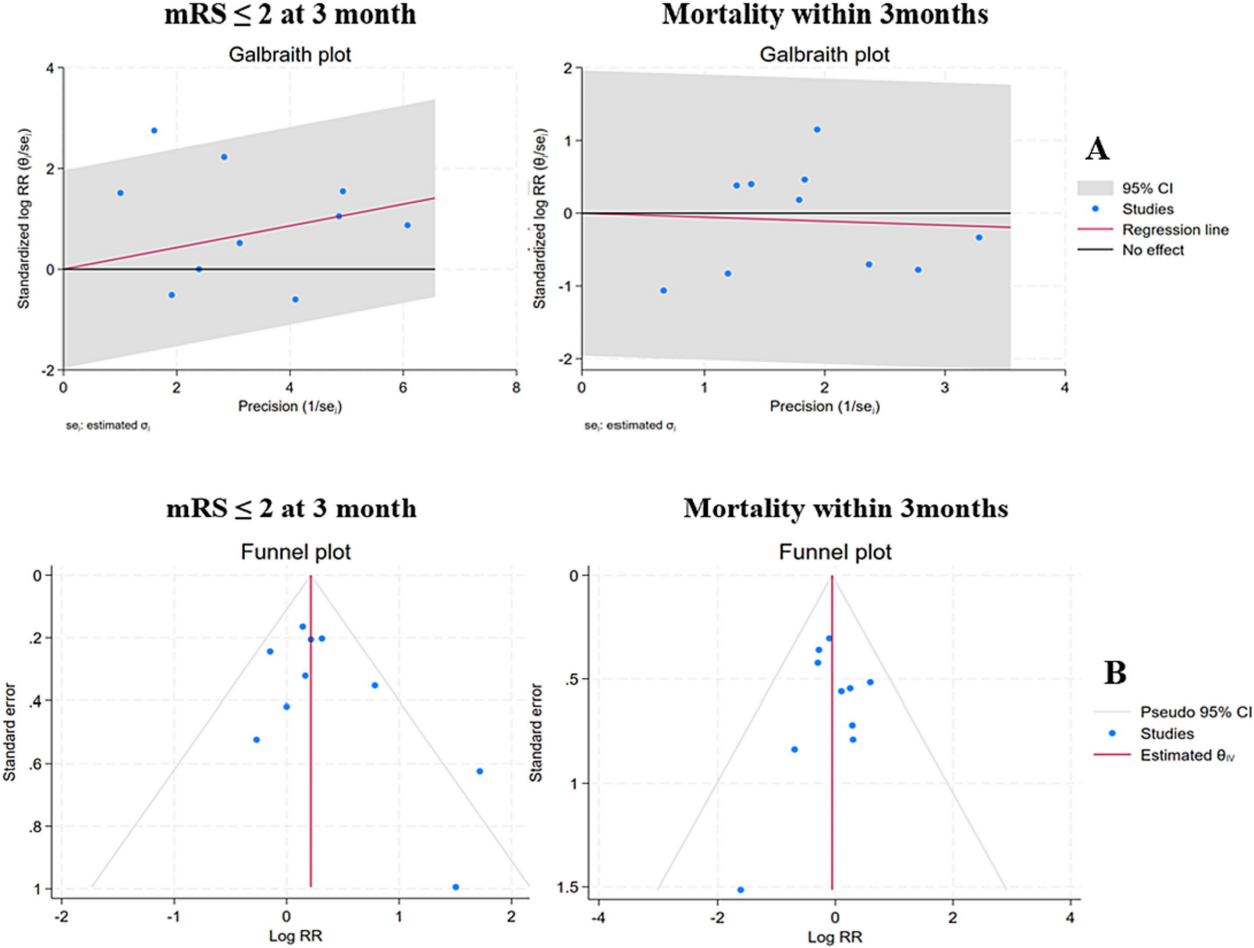
Galbraith plots and funnel plots of the studies used in the analysis of clinical outcomes. **(A)** Galbraith plots were employed to analyze the heterogeneity in the present meta-analysis. **(B)** The funnel plots of mRS and mortality were symmetrical, suggesting that the likelihood of publication bias was less likely.

As illustrated in Figure 2, the differences in the primary outcome between the hypothermia groups and the control groups were significant (RR = 1.28, 95% CI 1.01–1.61, *I*^2^ = 36%, *p* = 0.04, random-effects). Specifically, hypothermia treatment in conjunction with thrombolysis or mechanical thrombectomy was related to a mRS score of ≤2 at 3 months. Mortality within 3 months in TH groups was not significantly different from that in the control groups (RR = 0.95, 95% CI 0.69–1.29, *I*^2^ = 0.00%, *p* = 0.73, random-effects). Additionally, TH groups were not associated with an increased risk of pneumonia (RR = 1.35, 95% CI 0.76–2.40, *I*^2^ = 61%, *p* = 0.31, random-effects) and total complications (RR = 1.02, 95% CI 0.89–1.16, *I*^2^ = 33%, *p* = 0.77, random-effects). The *I*^2^ values for the mRS and mortality were 36 and 0% respectively, suggesting low heterogeneity (*I*^2^ < 50%). The Galbraith plot shows the same result (Figure 3A). Also, cardiac complications (RR 0.89, 95% CI 0.72–1.10, *I*^2^ = 0.00%, *p* = 0.29) and intracerebral hemorrhage (RR 1.02, 95% CI 0.81–1.28, *I*^2^ = 0.00%, *p* = 0.89) were not comparable between the two groups.

**Figure 4:**
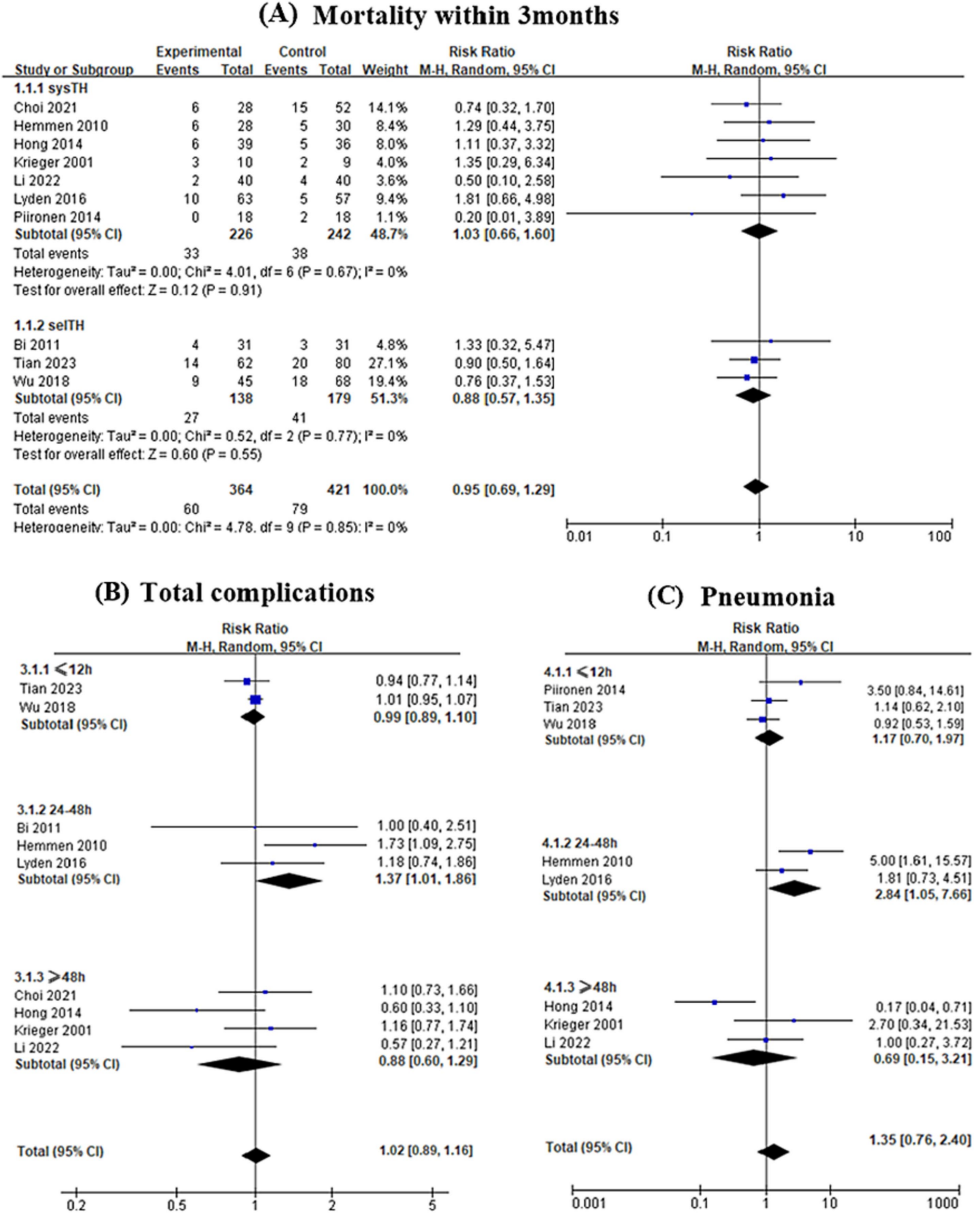
Subgroup analyses were performed based on hypothermia modality and duration. **(A)** The mortality between systemic hypothermia (systTH) and control groups was not statistically significant (RR = 1.03, 95% CI 0.66–1.60, *I*^2^ = 0%, *p* = 0.91). There was an indication of a mild protective effect of selective cerebral hypothermia (selTH), although the difference in mortality between selTH and control groups was not statistically significant (RR = 0.88, 95% CI 0.57–1.35, *I*^2^ = 0%, *p* = 0.55). Patients undergoing hypothermia for 24–48 h experienced a higher rate of total complications (RR = 1.37, 95% CI 1.01–1.86, *I*^2^ = 0%, *p* = 0.04) **(B)** as well as an increased incidence of pneumonia (RR = 2.84, 95% CI 1.05–7.66, *I*^2^ = 47%, *p* = 0.04) **(C)**.

Additionally, subgroup analyses were performed based on different study quality, hypothermia modality, and duration. Randomized studies reported a greater risk of pneumonia (RR 2.35, 95% CI 1.20–4.58, *p* = 0.01) in TH groups. Mortality (RCTs: RR 1.08, 95% CI 0.69–1.67, *p* = 0.74; OCTs: RR 0.80, 95% CI 0.52–1.23, *p* = 0.30), total complications (RCTs: RR 1.01, 95% CI 0.72–1.42, *p* = 0.94; OCTs: RR 1.00, 95% CI 0.95–1.06, *p* = 0.90) did not differ between TH groups and controls. The mortality between systTH and control groups was not statistically significant (RR = 1.03, 95% CI 0.66–1.60, *I*^2^ = 0%, *p* = 0.91). There was an indication of a mild protective effect of selTH, although the difference in mortality between selTH and control groups was not statistically significant (RR = 0.88, 95% CI 0.57–1.35, *I*^2^ = 0%, *p* = 0.55). Patients undergoing TH for 24–48 h experienced a higher rate of overall complications (RR = 1.37, 95% CI 1.01–1.86, *I*^2^ = 0%, *p* = 0.04) as well as an increased incidence of pneumonia (RR = 2.84, 95% CI 1.05–7.66, *I*^2^ = 47%, *p* = 0.04). However, when the duration of hypothermia was greater than 48 h, there were no significant differences in mortality and total complications between the hypothermia group and the control group (Figure 4).

### 3.4 Risk of publication bias assessment

Funnel plots were used to assess the possibility of publication bias, and the asymmetry of funnel plots was evaluated by Egger’s test. We considered significant publication bias as a *p*-value less than 0.1. An analysis of trim and fill was performed to further evaluate the potential existence of publication bias. In this analysis, Egger’s regression test showed no evidence of significant publication bias regarding mRS ≤2 at 3 months (*p* = 0.1392), mortality within 3 months (*p* = 0.9189), total complications (*p* = 0.8570), and pneumonia (*p* = 0.7814). The funnel plots showed symmetrical shapes, which suggested low publication bias (Figure 3B).

### 3.5 Sensitivity analyses

One study (17) was scored as a relatively lower quality based on the Newcastle–Ottawa Quality Assessment Scale. When this study was removed from the analysis, functional independence still did not differ between TH and controls (RR 1.21, 95% CI 0.95–1.55, *p* = 0.1).

## 4 Discussion

Although intravenous thrombolysis and thrombectomy can be effective in the treatment of ischemic stroke, there are still many patients with poor prognoses during treatment and recovery (20). The benefits of these therapies for stroke patients are limited by a variety of factors, such as time window and cerebral ischemia-reperfusion injury after revascularization (21). Consequently, finding ways to produce neuroprotection is of great importance.

Experimentally, hypothermia effectively ameliorated neuronal degeneration after global and focal brain ischemia in multiple models (22, 23). It might delay the depletion of energy reserves, alleviate cytoplasmic acidosis, slow the influx of calcium ions, inhibit the production of free oxygen radicals, and reduce the release of excitatory amino acids (24). A study (25) found that the cerebral infarction volume in mice was significantly smaller in the hypothermia combined with the recombinant tissue plasminogen activator (rtPA) group compared with rtPA alone. Studies using rodent models (26) showed that hypothermia, through various mechanisms, could effectively blunt the ischemic cascade. Hypothermia could decrease blood-brain barrier disruption and brain metabolism, alleviate inflammatory response, and inhibit apoptosis of cells in the ischemic brain (27). Overall, hypothermia is effective in reducing ischemic damage in experimental models (28).

Nevertheless, in clinical studies, some research has yielded differing conclusions regarding the prognosis of AIS patients with hypothermia treatment. One study (11) showed that ischemic stroke patients who received TH experienced significantly better outcomes compared to those who underwent conventional treatment. However, another study revealed (19) that no difference was observed between the two treatments for good neurologic outcomes. To elucidate the relationship between TH and prognosis in AIS patients, we conducted this meta-analysis. In this study, we found that hypothermia treatment was related to favorable functional outcomes (*p* = 0.04), and no significant publication bias was found using the Egger test (*p* = 0.1392).

Also, the efficacy and safety of a combination of TH and reperfusion therapy remain uncertain and should be further analyzed (29–32). In the present research, the authors conducted a meta-analysis to comprehensively assess the value of TH combining mechanical thrombectomy or thrombolysis in the treatment of ischemic stroke. Our findings indicated that there was no significant association between TH and the incidence of pneumonia, overall complications, and mortality within 3 months. However, in the subgroup analyses, there was an increased risk of overall complications and pneumonia in patients undergoing hypothermia for 24–48 h. Randomized studies reported a greater risk of pneumonia (RR 2.35, 95% CI 1.20–4.58, *p* = 0.01) in TH groups. Further well-designed prospective studies and RCTs with larger sample sizes are needed to validate the efficacy and safety of TH.

Induction of hypothermia can be achieved via systTH and selTH. Some studies (33, 34) confirmed that patients receiving systTH were potentially at higher risk for adverse effects due to decreased body temperature. There were several advantages to using selTH, such as rapid and effective induction, with negligible effects on core body temperature, and so on (35–37). In the present analyses, the mortality between systTH and control groups was not statistically significant. There was an indication of a mild protective effect of selTH, but the difference in mortality between selTH and control groups was not statistically significant (RR = 0.88, 95% CI 0.57–1.35, *I*^2^ = 0%, *p* = 0.55). The results may be attributed to the small number of patients in the two subgroups and the unequal sample size between the two subgroups.

Only one relevant meta-analysis (38) published in 2023, showed that TH was associated with favorable outcomes, independent of mortality and complications. Our study yielded results that were largely concordant with those of previous studies for the efficacy of therapeutic hypothermia. However, there were some discrepancies in the safety profile. Furthermore, the present study included subgroup analyses that were not available in previous studies. In addition, our research added to the literature (5) on the efficiency of combining mechanical thrombectomy with intraarterial selective cooling infusion published on March 24, 2023.

This study has several limitations. Firstly, the details of HT varied greatly among the included studies, potentially introducing heterogeneity. Despite the low heterogeneity in the synthesis, the small number of studies and patients included in this analysis limits its generalizability. Secondly, owing to the small number of RCTs available, we have to include observational cohort trials in this meta-analysis, which may affect the level of evidence and overall credibility. Nevertheless, the role of TH combined with reperfusion therapy appears to warrant further investigation given our findings. Based on these encouraging results and the limitations of the present study, a randomized clinical trial is ongoing to further evaluate the efficacy and safety of selective cerebral arterial hypothermia combined with endovascular treatment for acute anterior circulation large vessel occlusive cerebral infarction (Chinese Clinical Trial Registry, registration code: ChiCTR2400087155).

## 5 Conclusion

The preliminary evidence supports the safety and feasibility of hypothermia combined with reperfusion therapy, which should be further investigated in randomized controlled studies.

## Data Availability

The original contributions presented in the study are included in the article/supplementary material, further inquiries can be directed to the corresponding author.
