# Supplementary material for: The efficacy of hypothermia combined with thrombolysis or mechanical thrombectomy on acute ischemic stroke: a systematic review and meta-analysis
Source: Front Neurol. 2025 Jan 7;15:1481115. doi: 10.3389/fneur.2024.1481115 (PMC11746097; doi:10.3389/fneur.2024.1481115)
Supplement: Supplementary file 1 [file Data_Sheet_1.docx]

((((((((((Stroke*[Title/Abstract]) OR (Cerebrovascular Accident*[Title/Abstract])) OR (Cerebral Stroke*[Title/Abstract])) OR (Cerebrovascular Apoplexy[Title/Abstract])) OR (Brain Vascular Accident*[Title/Abstract])) OR (Cerebrovascular Stroke*[Title/Abstract])) OR (Apoplexy[Title/Abstract])) OR (Acute Stroke*[Title/Abstract])) OR (Acute Cerebrovascular Accident*[Title/Abstract])) OR ("Stroke"[Mesh])) AND ((("Infusions, Intra-Arterial"[Mesh]) OR ((((Intra-Arterial Infusion*[Title/Abstract]) OR (Intra Arterial Infusion*[Title/Abstract])) OR (Intraarterial Infusion*[Title/Abstract])) OR (Regional Arterial Infusion*[Title/Abstract]))) OR ((Hypothermia) OR (hypothermia* [Title/Abstract])))
