# Supplementary material for: The efficacy of hypothermia combined with thrombolysis or mechanical thrombectomy on acute ischemic stroke: a systematic review and meta-analysis
Source: Front Neurol. 2025 Jan 7;15:1481115. doi: 10.3389/fneur.2024.1481115 (PMC11746097; doi:10.3389/fneur.2024.1481115)
Supplement: Supplementary file 2 [file Data_Sheet_2.docx]

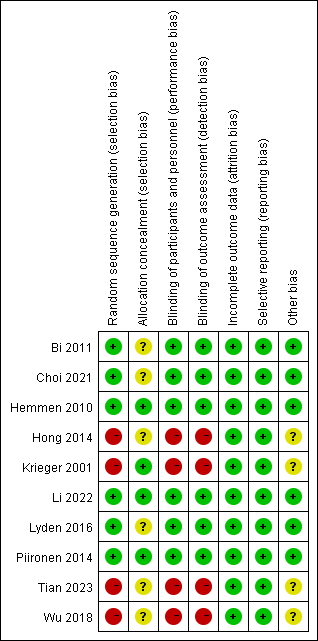


**Supplementary 2. Risk of bias summary**

We evaluated the risk of bias in the included trials according to the Cochrane Handbook for Systematic Reviews of Interventions. Red dots represent high risk, green dots represent low risk, and yellow dots represent unclear risk.
